# The Ameliorative Effects of the Ethyl Acetate Extract of *Salicornia europaea* L. and Its Bioactive Candidate, Irilin B, on LPS-Induced Microglial Inflammation and MPTP-Intoxicated PD-Like Mouse Model

**DOI:** 10.1155/2019/6764756

**Published:** 2019-07-09

**Authors:** Joonsoo Kim, Govindarajan Karthivashan, Mee-Hyang Kweon, Deuk-Hoi Kim, Dong-Kug Choi

**Affiliations:** ^1^Department of Applied Life Sciences, Graduate school of Konkuk University, Research Institute of Inflammatory Diseases, Chungju 27478, Republic of Korea; ^2^Research Center, Phyto Corporation, Seoul 08826, Republic of Korea; ^3^Department of Integrated Bioscience and Biotechnology, College of Biomedical and Health Science, Nanotechnology Research Center, Konkuk University, Chungju 27478, Republic of Korea

## Abstract

Hyperactivation of microglia, the resident innate immune cells of the central nervous system, exacerbates various neurodegenerative disorders, including Parkinson's disease (PD). Parkinson's disease is generally characterized by a severe loss of dopaminergic neurons in the nigrostriatal pathway, with substantial neuroinflammation and motor deficits. This was experimentally replicated in animal models, using neurotoxins, i.e., LPS (lipopolysaccharides) and MPTP (1-methyl-4-phenyl-1,2,3,6-tetrahydropyridine). *Salicornia europaea* L. (SE) has been used as a dietary supplement in Korea and Europe for several years, due to its nutritional and therapeutic value. In this study, we intend to investigate the antineuroinflammatory and anti-PD-like effects of the bioactive fraction/candidate of the SE extract. Initially, we screened various fractions of SE extract using an *in vitro* antioxidant assay. The optimal fraction was investigated for its *in vitro* antineuroinflammatory potential in LPS-stimulated BV-2 microglial cells and *in vivo* anti-PD-like potential in MPTP-intoxicated mice. Subsequently, to identify the potential candidate responsible for the elite therapeutic potential of the optimal fraction, we conducted antioxidant activity-guided isolation and purification; the bioactive candidate was structurally characterized using nuclear magnetic resonance spectroscopy and chromatographic techniques and further investigated for its *in vitro* antioxidative and antineuroinflammatory potential. The results of our study indicate that SE-EA and its bioactive candidate, Irilin B, effectively alleviate the deleterious effect of microglia-mediated neuroinflammation and promote antioxidative effects. Thus, they exhibit potential as therapeutic candidates against neuroinflammatory and oxidative stress-mediated PD-like neurodegenerative complications.

## 1. Introduction

Parkinson's disease (PD), the second most commonly reported neurodegenerative disease (NDD), is clinically characterized by the progressive loss of dopaminergic neurons in the basal ganglia regions (i.e., precisely at the substantia nigra pars compacta (SNpc) and striatum (STR)) [1, 2]. Although the etiological factors of PD are unclear, the progression of the disease is linked to the impairment of reactive oxygen species- (ROS-) defensive mechanisms, protein misfolding and aggregate formation, aberrant hyperactivation of microglial cell-mediated inflammatory cascades, and subsequent functional motor deficits (i.e., bradykinesia, postural instability, and resting tremors) [2, 3]. At the molecular level, ROS-mediated DNA damage, protein dysfunction, and lipid peroxidation eventually lead to detrimental cell death. Accumulating evidence suggests that the redox imbalance actively facilitates the progression of neurodegenerative PD [3–5]. The heme oxygenase (HO) enzyme complex is one of the most commonly reported physiological defensive antioxidant systems. In particular, the heme oxygenase-1 (HO-1), a stress-inducible heat shock protein, is triggered by various oxidative and inflammatory signals and actively participates in the breakdown of free heme radicals into cytoprotective byproducts, thereby retaliating to the oxidative conditions [6, 7]. In fact, the upsurge of HO-1 expression has been observed to facilitate an adaptive cell survival response against inflammation and oxidative injury [8]. The immunohistochemical post-mortem reports on the brains of PD patients show that the brains exhibit substantial expression of HO-1-positive stains, which indicates the role of HO-1 in PD pathogenesis [9]. On the other hand, microglia-mediated neuroinflammation is one of the major pathological hallmarks of several NDD complications, including PD [10]. Microglial cells are resident macrophages, predominantly involved in maintaining cell homeostasis and the functional integrity of the central nervous system (CNS) by providing an effective first-line defense mechanism [10, 11]. Microglial cells are reported to actively participate in the phagocytic clearance of dead neuronal cells and are also known to help in reviving neuronal cells by releasing several neurotropic factors [12]. However, the pivotal role of glial cells involves encountering and removing exogenous pathogens, toxins, and other foreign bodies that enter the CNS, thereby preventing disease-mediated pathological events. Alternatively, evidence suggests that the glial cell activation is also aggravated by exogenous factors and the progression of the disease cascade [13, 14]. For instance, in the case of PD, the ROS insult, protein aggregate accumulation, and mitochondrial dysfunction-mediated stimulation of glial cells potentially activate the canonical nuclear factor kappa-light-chain-enhancer (NF-*κ*B) and subsequent mitogen-activated protein kinase (MAPK) pathways. This event leads to release of several proinflammatory cytokines, i.e., tumour necrosis factor (TNF-*α*), interleukins (IL-1*β* and IL-6), and proinflammatory mediators, i.e., nitric oxide (NO), inducible nitric oxide synthase (iNOS), and cyclooxygenase-2 (COX-2) [15, 16]. These cascades eventually result in dopaminergic cell death and neuronal loss in the nigrostriatal regions, as has been observed in post-mortem brain reports of PD patients [17, 18]. This nigrostriatal depletion of dopamine and dopaminergic neurons has been well documented to attribute to the tremors, bradykinesia, and other motor function defects in PD patients [18, 19]. Experimentally, the ROS and neuroinflammatory cascades of PD can be substantially replicated by lipopolysaccharide- (LPS-) stimulated BV-2 microglial cells (*in vitro*) and 1-methyl-4-phenyl-1,2,3,6-tetrahydropyridine (MPTP) when inflicted on PD-like animal models, to an extent [20–22]. In particular, MPTP-treated mice exhibit several significant clinical hallmarks of PD pathogenesis, such as neuroinflammation and loss of tyrosine hydroxylase- (TH-) positive neurons, specifically in the striatal and SNpc regions, and substantial motor impediments (including tremors and postural instability) [21]. Thus, several studies have adapted these models to investigate the anti-PD-like therapeutic potential of various bioactive candidates [21, 23, 24].

*Salicornia europaea* L. (SE; synonym is *S. herbacea*) is commonly known as *glasswort* in English and *ham-cho* in Korean. It is widely distributed in salt marshes throughout coastal regions across the Mediterranean and East Asia [25, 26]. The leaves of this plant are commonly harvested and consumed by locals as a salt substitute, raw vegetable, and also as a nutritious fermented food in Korea and various European countries [26, 27]. Because the plant grows in extreme saltwater habitats, it produces several defensive secondary metabolite compounds to sustain “salt stress”; these include flavonoids, saponins, and alkaloids [27, 28]. Much scientific evidence has reported the antioxidative, antitumour, antiadipogenic, antidiabetic, and neuroprotective potential of SE, which can potentially be attributed to the plant's secondary metabolites [27–31]. However, in this study, we intend to investigate the antineuroinflammatory and anti-PD-like effects of the bioactive fraction (SE-EA) and bioactive candidate, Irilin B, of desalted *Salicornia europaea* in LPS-stimulated BV-2 microglial cells and MPTP-intoxicated PD-like mice. To the best of our knowledge, this is the first study to report the antineuroinflammatory potential of Irilin B.

## 2. Materials and Methods

### 2.1. Reagents

Sodium nitrite, N,N-dimethyl-1-naphthylamine, lipopolysaccharide (LPS) (*E. coli* 055:B5), 2′,7′-dichlorofluorescin diacetate (DCFDA), thiazolyl blue tetrazolium bromide (MTT), and 1-methyl-4-phenyl-1,2,3,6-tetrahydropyridine (MPTP) hydrochloride were purchased from Sigma-Aldrich (St. Louis, MO, USA). A 10x RIPA buffer was obtained from Millipore (Milford, MA, USA), and protease and phosphatase inhibitor cocktail tablets were purchased from Roche (Indianapolis, IN, USA). Dimethyl Sulfoxide (DMSO) was provided from Amresco (Solon, OH, USA), and phosphoric acid was obtained from Duksan Pure Chemicals (Ansan-si, Gyeonggi-do, Korea).

### 2.2. SE Extraction and Bioactivity-Guided Fractionation

Initially, the desalted SE hot water extracts were prepared and subjected to fractionation using different polarity-based solvent partitions; the obtained fractions were compared and screened based on their phytochemical profiles and *in vitro* antioxidant potential.

#### 2.2.1. Preparation of Desalted SE Hot Water (SE-HW) Extracts: Fractionation and Phytochemical Profiling of SE-Fractions

**Figure 1:**
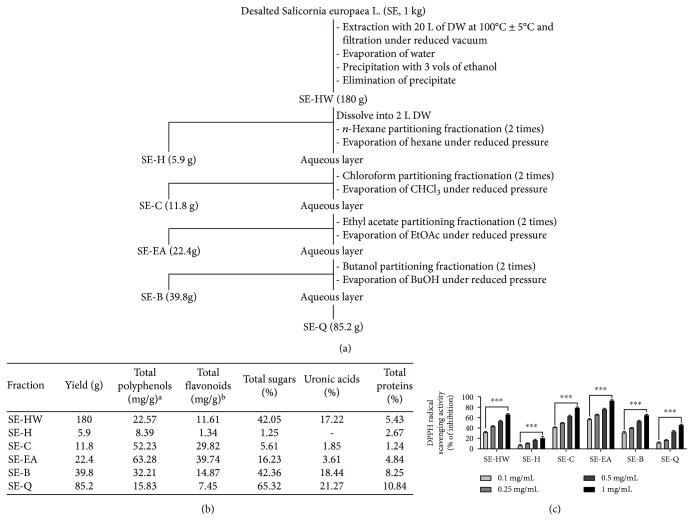
Phytochemical profiling and antioxidant screening of varied SE subfractions. Fractionation of *Salicornia europaea* L. hot water (SE-HW) extract using organic solvent partitioning. Chemical compositions and antioxidative activities of the SE subfractions obtained from organic solvent partitioning of SE-HW. We yield SE-HW's fraction of *n*-hexane (SE-H), chloroform (SE-C), ethyl acetate (SE-EA), butanol (SE-B), and remained (SE-Q). (a) Organic solvent partitioning of SE-HW, (b) chemical composition of 6 subfractions, and (c) antioxidant activities of SE subfractions obtained from organic solvent partitioning of *Salicornia europaea* hot water (SE-HW) extract. Antioxidative activity was measured using DPPH radical scavenging potential (c). ^∗∗∗^ *p* < 0.001 vs. lowest concentration.

The stems and leaves of *Salicornia europaea* L. (SE) were acquired from the western seashore marshes of South Korea and were registered as voucher specimens deposited at the R&D centre of the Phyto Corporation (Seoul National University, South Korea). The extraction and fractionation procedure of the SE is depicted in Figure 1(a). In brief, one kilogram of SE plant material was macerated in 20 L of distilled water at 100 ± 5°C and condensed in a vacuum. Subsequently, the precipitate was exposed to 3 volumes of ethanol, and the formed precipitate was eliminated to obtain the hot water extracts of SE (SE-HW). A 180 g of SE-HW was dissolved in 2 L of distilled water and subsequently partitioned using a variety of polarity-based organic solvents, including *n*-hexane, chloroform, ethyl acetate, and *n*-butanol. Each partitioning was performed twice to ensure that the potential yield of fractions and the obtained solvent partitions were condensed under pressure to obtain the respective hexane (SE-H, 5.9 g), chloroform (SE-C, 11.8 g), ethyl acetate (SE-EA, 22.4 g), and butanol (SE-B, 39.8 g) fractions. The remaining precipitate was also further condensed and processed as described above to obtain SE-Q (85.2 g) fractions.

The nutritional information about total polyphenol, flavonoids, sugars, uronic acids, and proteins of *Salicornia europaea* L. subfractions is presented in Figure 1(b). The total carbohydrate (sugar) and uronic acid contents in the SE extract and fractions were assessed using the phenol-sulphuric and *p*-hydroxy diphenyl methods, respectively [32, 33]. The protein content was measured using the method proposed by Lowry et al. (see [34]). The total phenolic content was determined using Folin-Ciocalteu reagents, as previously described, with slight modifications [35]. Briefly, the SE extract and fractions or gallic acid standard (20 *μ*L) were mixed with 250 *μ*L of 2% sodium bicarbonate and incubated at room temperature for 5 min. Then, 16 *μ*L of 50% Folin-Ciocalteu reagent (prediluted 2-fold with distilled water) was added to the mixture. After 30 min incubation at room temperature, their respective absorbance was measured at 725 nm. Aqueous solutions of known gallic acid concentrations in the range of 10-500 *μ*g/mL were used for calibration. The results are expressed as mg gallic acid [36] equivalent (GAE mg)/g samples. Total flavonoid content was measured using the Abdel-Hameed method, with slight modifications. Briefly, samples or rutin standard dissolved in methanol (20 *μ*L) was mixed with 200 *μ*L of 2% diethylene glycol and allowed to incubate at 30°C for 60 min. Afterward, 6 *μ*L of 1 N NaOH solution was added to the mixture. After a 10 min incubation at room temperature, absorbance was measured at 420 nm. Methanolic solutions of known rutin concentrations in the range of 10-1000 *μ*g/mL were used for calibration, and the results were expressed as mg rutin equivalent (REQ mg)/g samples.

#### 2.2.2. Antioxidant Activity-Based Screening of Bioactive Fractions

Antioxidant activities of SE-HW and its subfractions were determined using a 2,2-diphenyl-1-picrylhydrazyl- (DPPH-) radical scavenging assay based on its scavenging potential of stable DPPH-free radicals, as described earlier, but with slight modifications [37]. The SE extracts and solvent fractions were dissolved in methanol (20 *μ*L) and then incubated with 200 *μ*M DPPH (Sigma) ethanolic solution (180 *μ*L) at 37°C for 30 min in a 96-well microtiter plate. After the incubation period, the respective absorbance of the reaction mixtures was subsequently measured at 517 nm, and the percentage of inhibition and IC_50_ values were calculated.

### 2.3. Antineuroinflammatory and Anti-ROS Potential of SE-EA

The chosen optimal bioactive fraction was further investigated for its *in vitro* antineuroinflammatory and anti-ROS potential in LPS-induced BV-2 microglial cells.

#### 2.3.1. Cell Culture and Treatment

The BV-2 microglial cells were cultured and maintained at 37°C, with 5% of CO_2_, in a humidified incubator (Panasonic, Osaka, Japan). As previously noted (38), the cells were provided with Dulbecco's modified Eagle's medium (DMEM) (Gibco, Carlsbad, CA, USA) and supplemented with 5% of fetal bovine serum (FBS), 50 units/mL of penicillin, and 50 *μ*g/mL of streptomycin (purchased from Gibco). They were maintained in a 100 mm tissue culture dish (Falcon, Oneonta, NY, USA). For the experiments, the cells were seeded at respective densities on 6-, 24-, or 96-well culture plates (SPL, Pocheon-si, Gyeonggi-do, Korea). When the microglial cells reached 70~80% confluence, the cells were pretreated for 1 h with various concentrations of SE-EA (20, 100, and 200 *μ*g/mL) or Irilin B (2, 10, and 20 *μ*M), followed by an LPS (200 ng/mL) treatment for 20 h.

#### 2.3.2. Cell Viability, NO Release, and DCFDA Intracellular ROS Assay

The cell viability of BV-2 cells after administration of the SE-EA and Irilin B was determined by the MTT reagent assay, as reported earlier. In brief, the BV-2 cells, which were seeded in 96-well plates (5 × 10^4^ cells/well), were treated with LPS (200 ng/mL) in the presence or absence of SE-EA (20, 100, and 200 *μ*g/mL) or Irilin B (2, 10, and 20 *μ*M) for 20 h and subsequently treated with an MTT reagent solution and incubated for 1 h at 37°C. Furthermore, the supernatant was discarded and the insoluble MTT formazan crystals were dissolved with DMSO; their corresponding absorbance was measured at 540 nm using a sunrise spectrophotometer (Tecan, Grödig, Austria).

The inhibition effects of SE-EA and Irilin B on biosynthesized NO in BV-2 cells stimulated by LPS was determined by the Griess reagent assay, as previously reported. In brief, the BV-2 cells were seeded in 24-well culture plates, and after reaching a confluence of 70%, the cells were incubated with LPS (200 ng/mL) in the presence or absence of SE-EA (20, 100, and 200 *μ*g/mL) or Irilin B (2, 10, and 20 *μ*M) for 20 h at 37°C in a supplied humidified incubator with 5% CO_2_. Subsequently, the supernatants were transferred to 96-well plates and exposed to an equal volume of the Griess reagent. Their corresponding absorbance was measured at 552 nm using the sunrise spectrophotometer (Tecan, Grödig, Austria). The NO production was calculated using a predetermined sodium nitrite standard solution.

The anti-ROS potential of SE-EA and Irilin B in the LPS-induced BV-2 cells was determined by quantifying the relative internal ROS generation using DCF fluorescence, according to the manufacturer's protocol. In brief, the BV-2 microglial cells were seeded onto 96-well culture plates. The cells were incubated in the presence or absence of SE-EA (20, 100, and 200 *μ*g/mL) or Irilin B (2, 10, and 20 *μ*M) for 20 h at 37°C in a supplied humidified incubator with 5% CO_2_. Furthermore, a DCFDA solution was added to each well, which were then incubated for 30 mins. After the incubation period, the cells were washed twice with PBS; the relative DCF fluorescence intensity was measured using the SpectraMax M2 (Molecular Devices, San Jose, CA, USA; excitation (Ex)/emission (Em), 485/538 nm).

### 2.4. Quantification of Proinflammatory Cytokines/Mediators and Antioxidant Biomarker

The antineuroinflammatory and anti-ROS potential of the optimal SE-EA fraction and the bioactive Irilin B was further quantified at the molecular level by investigating the modulation of the protein- and RNA-level expression of the proinflammatory cytokines/mediators, as well as the protein levels of the stress-inducible heat shock protein, HO-1, using western blot and quantitative real-time polymerase chain reaction (qRT-PCR) techniques.

#### 2.4.1. Western Blot Analysis

BV-2 cells, pretreated with SE-EA (20, 100, and 200 *μ*g/mL) or Irilin B (2, 10, and 20 *μ*M), were inflicted with LPS (200 ng/mL) and incubated for 20 h. The treated cells were washed twice with PBS and lysed using a 1x RIPA buffer (Millipore, CA, USA) and a phosphatase and protease inhibitor cocktail (Roche, Mannheim, Germany) for 10 min at 4°C. Subsequently, the obtained cell lysates were centrifuged at 14,000 rpm, at 4°C, and the corresponding supernatants were collected and stored in separate vials for further analysis. Prior to electrophoresis, the protein content of the supernatant was quantified using a DC Protein Assay kit (Bio-Rad) and an equal amount of protein concentration (20-40 *μ*g) was separated according to the molecular weights in a 10% sodium dodecyl sulfate-polyacrylamide electrophoresis gel for each sample. This was then carefully transferred to the polyvinylidene difluoride membranes (Millipore, Bedford, MA, USA). The membranes were preincubated in 3% BSA (TBS buffer) for 1 h and incubated with the respective primary antibodies: anti-iNOS (1 : 2,000; BD Bioscience, CA. USA), anti-COX-2 (1 : 2,000), and anti-*β*-actin (1 : 2,000; Santa Cruz Biotechnology, TX, USA), rocking overnight at 4°C. On the following day, the membranes—bounded with the primary antibodies—were washed with TBS and incubated with species-specific horseradish peroxidase-conjugated-specific secondary antibodies (1 : 2000; Cell Signaling Technology, MA). The specific bands were detected using the PowerOpti-ECL kit, and the immunoblots bands were visualized by the Animal Genetics Inc. (Gyeonggi-do, Korea) detection system, according to the manufacturer's instructions. The bands were quantified using ImageJ software (National Institutes of Health, Bethesda, MD, USA).

#### 2.4.2. Total RNA Isolation and Quantitative Real-Time Polymerase Chain Reaction (qRT-PCR) Analysis

The RNA was isolated from the BV-2 cells treated with LPS (200 ng/mL) in the presence or absence of SE-EA (20, 100, and 200 *μ*g/mL) or Irilin B (2, 10, and 20 *μ*M) for 6 h, using the TRIzol reagent (Invitrogen Life Technologies, Carlsbad, CA, USA), as per the manufacturer's instructions. Total RNA isolation was quantified using the SpectraMax QuickDrop (Molecular Devices, San Jose, CA, USA). Initially, 2500 ng of total RNA and the GoScript™ Reverse Transcription System (Promega, USA) were utilized to obtain the cDNA; subsequently, a qRT-PCR was performed (denaturation: 30 sec at 95°C, annealing: 30 sec at 55°C, and extension: 72°C for 30 sec (50 cycles)). The sequences used for the primers targeting proinflammatory genes are listed in Table 1. The amplified product for each gene was run on electrophoresis on a 1% agarose gel, followed by ethidium bromide staining. The bands of the gels were photographed, and the respective band intensities were quantified using ImageJ software (National Institutes of Health, Bethesda, MD, USA) and normalized to the mRNA band intensity of glyceraldehyde 3-phosphate dehydrogenase (GAPDH). The results are representative of three independent experiments.

### 2.5. Investigation on Functional Motor Deficits and the Levels of TH Staining in the Mouse Model

To further investigate the anti-PD-like potential of the SE-EA fraction, we investigated the effects of SE-EA on MPTP-inflicted PD-like mice. The functional motor coordination deficit was determined through pole tests, and the tyrosine hydroxylase-expressing neurons were immunohistochemically stained and quantified to determine its therapeutic effects.

#### 2.5.1. Animal Housing and Experimental Groups

Male C57BL/6 mice (*n* = 9/group, age: 6-7 weeks, and weight: 26 ± 3 g) were obtained from DBL (Chungcheongbuk-do, Korea). The animals were housed in a controlled environment (23 ± 1°C and 50 ± 5% humidity) with 12 h dark-light cycles and permitted food and water *ad libitum*. Prior to the start of the experiment, all of the animals were allowed an acclimation period of 1~2 weeks. The experiment was conducted over the course of 7 days, and the animals were divided into four groups (sham, negative control (MPTP), MPTP+SE-EA 50, and MPTP+SE-EA 100). The SE-EA powder was dissolved in a 0.9% isotonic saline and administered orally for 7 days using a feeding catheter and sterilized syringe. The neurotoxin MPTP (20 mg/kg/2-hour intervals) was administered only on Day 2, four times at 2 h intervals. The sham group was treated with only a 0.9% isotonic saline during the treatment period. All of the animal experiments were performed in accordance with the Principles of Laboratory Animal Care (NIH publication no.85-23, revised 1985) and approved by Konkuk University Institutional Animal Care and Use Committee (KU17023).

#### 2.5.2. Behavioural-Pole Test

A pole test is a behavioural study intended to determine the PD-like bradykinesia symptoms in mice. The test was conducted on Day 7, as previously noted. For this test, each animal was placed at the top of a rough-surfaced pole (8 mm diameter and 55 cm height), with a heads-up posture. The time taken by the animal to turn at the top of the pole (time of turn) and the time taken by the animal to reach the bottom of the pole (time of descent) were noted. The period of time taken by the animal to both turn and descend relatively reflects the bradykinesia parameter. The test was conducted three consecutive times for each animal.

#### 2.5.3. Immunohistochemistry (IHC) and Image Analysis

After the pole test, the animals were anesthetized with a 23% urethane solution (Sigma-Aldrich, CA, USA), for the purpose of immunohistochemical investigations. As described earlier, followed by a saline flush, individual mouse's brains were perfused with a 4% paraformaldehyde (PFD) solution (Biosesang, Gyeonggi-do, Korea) via a cardiac puncture. The excised brains were further fixed at 4°C in the same PFD fixative and subsequently dehydrated in a 30% sucrose solution. The brains were then embedded in a tissue-freezing medium (Leica GmbH, Heidelberger, Germany) and sectioned coronally to obtain the striatal and SNpc regions. The free-floating sections were subsequently incubated with specific anti-TH (Calbiochem, Darmstadt, Germany). Antigen-positive areas were visualized using a VECTASTAIN ABC kit and a DAB Peroxidase (HRP) Substrate Kit (Vector Laboratories, CA, USA). The IHC stained slides were digitized using a Nikon Eclipse Ts2 microscope (Nikon, Tokyo, Japan). The digitized slide images were processed and analysed using the “analyse particle” function of ImageJ (NIH, MD, USA).

### 2.6. Bioactivity-Guided Isolation and Characterization of Irilin-B from SE-EA Fraction

Furthermore, we extended our study to identify the possible candidate responsible for the enhanced bioactivity of the SE-EA fraction. In accordance, the compounds of the SE-EA fraction were separated and isolated using chromatographic techniques and simultaneously screened for their bioactivity based on *in vitro* DPPH radical scavenging potential.

#### 2.6.1. Antioxidant Activity-Guided Isolation, Purification, and Characterization

**Figure 2:**
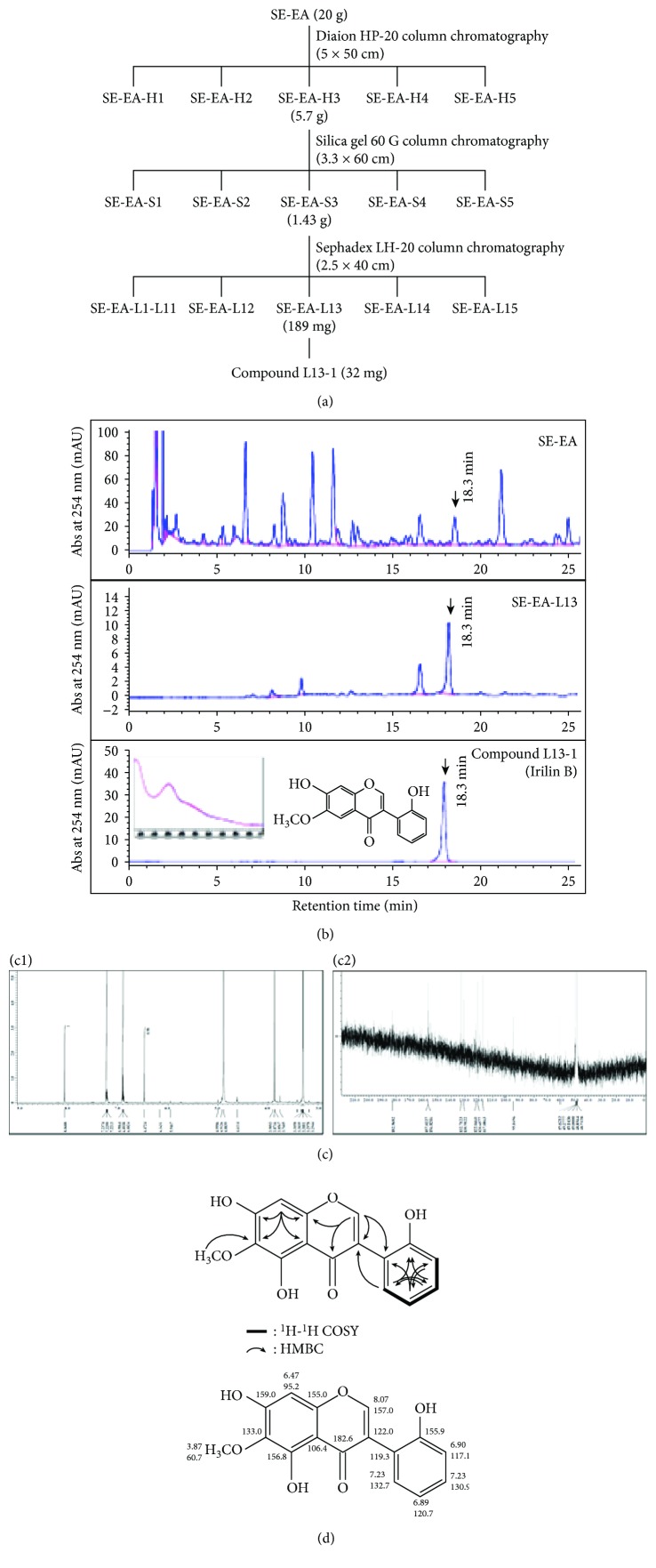
Bioactivity-guided isolation and characterization of Irilin-B from SE-EA fraction. Antioxidant activity-guided isolation and characterization of compound L13-1 from SE-EA. (a) Purification scheme of compound L13-1 from SE-EA; (b) representative HPLC profiles of SE-EA, SE-EA-L13, and finally purified compound L13-1, Irilin B. HPLC (1260 Infinity, Agilent Technologies, Santa Clara, CA, USA) equipped with a ZORBAX Eclipse XDB C18 prep column (9.4 × 250 mm, 5 *μ*m, Agilent Technologies) was conducted with a gradient eluent of methanol and 0.04% trifluoroacetic acid as the mobile phase. The UV spectrum and chemical structure of the finally purified compound L13-1 are also depicted. (c) NMR spectra of the purified compound L13-1. c1: ^1^H-NMR spectrum; c2: ^13^C-NMR spectrum. (d) Determination of stereographic structure by analysis of two-dimensional ^1^H-^1^H COSY and HMBC-NMR spectra and assignments of carbon and hydrogen in NMR spectra.

The schematic outline of the compound isolation from SE-EA is depicted in Figure 2(a). In brief, 20 g of the SE-EA fraction was separated through HP-20 column chromatography to obtain several subfractions, which were screened based on the DPPH antioxidant assay, as reported in Section 3.2.2. Then, the obtained elite fraction was separated by Silica gel 60 G column and Sephadex LH-20 column, by screening the obtained subfractions using an antioxidant assay as reported above. The HPLC (1260 Infinity, Agilent Technologies, Santa Clara, CA, USA) equipped with a ZORBAX Eclipse XDB C18 prep column (9.4 × 250 mm, 5 *μ*m, Agilent Technologies) was conducted with a gradient eluent of methanol and 0.04% trifluoroacetic acid as the mobile phase (Figure 2(b)). The final candidate exhibiting enhanced antioxidant potential was identified using nuclear magnetic resonance (NMR) studies to interpret its spectra and confirm its chemical structure (Figure 2(d)).

### 2.7. Effects of Irilin B in Molecular Level Alterations of Proinflammatory Cytokines/Mediators and Antioxidant Biomarker

The compound, Irilin B, exhibited enhanced *in vitro* antioxidant potential, which was further confirmed for its therapeutic potential using *in vitro* antineuroinflammatory and anti-ROS potential in LPS-induced BV-2 microglial cells, as described in Supplementary Figure 1(a).

### 2.8. Statistical Analysis

The data were analysed using a one-way ANOVA, followed by Tukey's Honest Significant Difference test (GraphPad Prism 5, GraphPad Software). The accepted difference values for statistically significant *p* values are reported in the legends of the corresponding figures. All of the results are presented as the mean ± SD.

## 3. Results and Discussion

Neuroinflammation-mediating neuronal cell death is one of the major clinical hallmarks of several neurodegenerative disorders (NDD), such as AD and PD. Under the diseased pathological conditions of NDDs, infection and alteration in the microenvironmental factors have been reported to dynamically participate in the hyperactivation of microglial cells, and it results in neuronal cell-death cascades which mediate disease progression [38, 39]. Although the activation of glial cells has also been conversely reported to protect and enhance the neuronal repair process, the pathogenic deterioration of the disease conditions creates an imbalance in this double-edged sword, like the functions of glial cells [16, 40]. The detrimental effect of hyperactivated microglia and astrocytes inflicts chronic inflammation on the neuronal milieu by elevating the proinflammatory cytokines—TNF-*α*, IL-1*β*, and IL-6—and mediators—iNOS and COX-2. This insult further provokes the excessive production of cytotoxic NO, superoxide, and ROS conditions [39, 41, 42]. As the brain is highly sensitive to oxidative conditions, the excessive ROS and imbalance in mitochondrial redox levels disrupt the integrity and function of neuronal cells [41, 43]. Precisely in the case of PD, the post-mortem brain reports of PD patients show that the brains exhibit elevated oxidative stress, evidenced by the means of extensive protein oxidation at the SNpc regions compared to the brains of healthy subjects. The level of glutathione (GSH, an antioxidant enzyme) was observed to be exhausted in the surviving neurons in the SNpc regions of PD patients [44]. Thus, curbing the neuroinflammation and retaliating oxidative stress in the brain milieu are considered effective strategies to alleviate symptomatic PD-like NDDs [45]. Accumulating evidence has reported that LPS (a direct microglial-mediated inflammatory stimuli) and MPTP (a neurotoxin-mediated microgliosis) substantially develop PD-like neuroinflammation and progressive neurodegeneration in experimental animal models [46, 47]. Recently, various extracts of SE-EA have been reported to exhibit therapeutic effects against a wide range of clinical complications, such as antidiabetic, antihypertension, antihyperlipidemia, and hepatoprotective complications [27, 48]. Thus, in this study, the antineuroinflammatory, antioxidative, and anti-PD-like potential of SE-EA/bioactive candidate was investigated in the aforementioned experimental models.

### 3.1. Phytochemical Profiling and Antioxidant Screening of Varied SE Subfractions

One-kilogram dry weight of desalted SE extract yields 180 g of hot water extracts (SE-HW); the solvent-solvent partitioning of SE-HW yielded 5.9 g of SE-H, 11.8 g of SE-C, 22.4 g of SE-EA, 39.8 g of SE-B, and 85.2 g of SE-Q, as depicted in Figure 1. According to the phytochemical profiling data, the SE-ethyl acetate (SE-EA) fraction indicates maximum total phenolic and total flavonoid content, with 63.28 and 39.74 mg/g, respectively, followed by SE-chloroform fraction (SE-C) with 32.21 and 14.87 mg/g, respectively; the SE-hexane (SE-H) fraction scored the least with 8.39 and 1.34 mg/g, respectively. However, among the SE-fractions—excluding the SE-Q fraction—the SE-butanol fraction exhibited the highest total carbohydrates, total uronic acid, and total protein contents, with 42.36%, 18.44%, and 8.25%, respectively, followed by SE-ethyl acetate fraction with 16.23%, 3.61%, and 4.84%, respectively. The DPPH antioxidant activity-based screening of the SE-HW and its fractions exhibited SE-EA as the elite bioactive fraction with the lowest IC_50_ value of 0.06 ± 0.01 mg/mL, followed by SE-C with 0.27 ± 0.01 mg/mL, and SE-B with 0.56 ± 0.01 mg/mL (Figure 1(c)). The least activity was exhibited by SE-H with 2.79 ± 0.01 mg/mL. The order of antioxidant activities and phenolic/flavonoid content is presented as follows:

*Order of antioxidant activities*: SE − EA > SE − C > SE − HW > SE − B > SE − Q > SE − H

*Order of total phenolic/flavonoid content*: SE − EA > SE − C > SE − B > SE − HW > SE − Q > SE − H

### 3.2. Antineuroinflammatory and Anti-Parkinsonism-Like Potential of SE-EA

#### 3.2.1. The Cytotoxicity Effects and NO Inhibitory Potential of SE-EA in LPS-Stimulated BV-2 Microglial Cells

**Figure 3:**
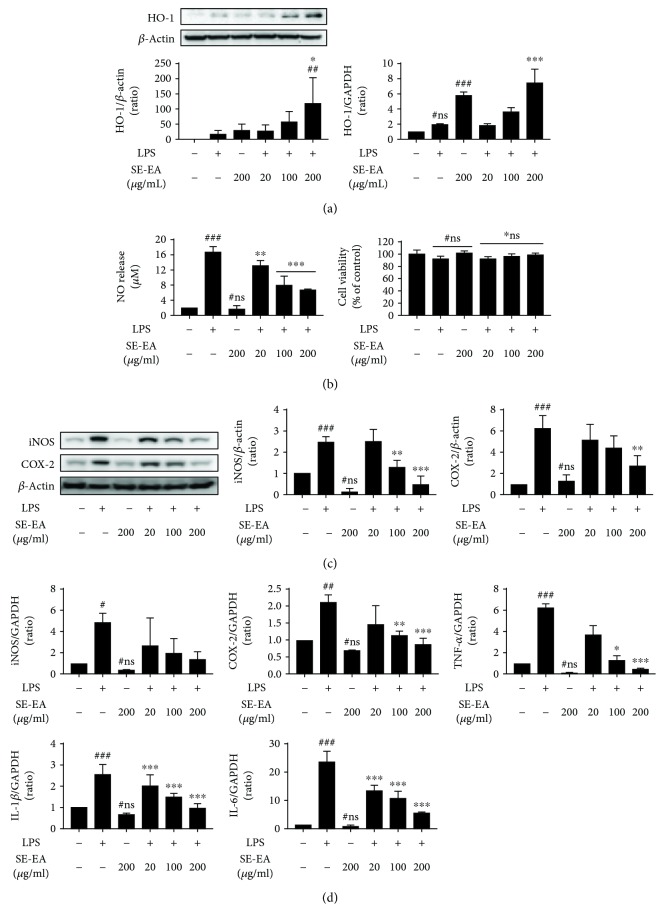
The cytotoxicity effects and NO inhibitory potential of SE-EA in LPS-stimulated BV-2 microglial cells. SE-EA (20,100, and 200 *μ*g/mL) were treated onto BV-2 cells with or without LPS and incubated in a CO_2_-supplied incubator for 20 hours. A ROS defense protein and HO-1 expression levels were analysed by a western blot and qRT-PCR (a). SE-EA treatment onto BV-2 microglial cells reduced ROS levels and induced HO-1 expressions. Anti-inflammatory effects of SE-EA in LPS-stimulated BV-2 microglial cells. SE-EA (20, 100, and 200 *μ*g/mL) and LPS (200 ng/mL) were cotreated onto BV-2 cells, which were incubated for 20 hours in a CO_2_-supplied incubator. Each group's nitric oxide release was measured using a Griess reagent, and cell viability was assayed by an MTT reagent (b). Of the western blot analysis, inflammatory mediators iNOS and COX-2 expression levels were presented. The intensity of each protein band was measured using ImageJ (c). The expressions of iNOS, COX-2, and proinflammatory cytokines TNF-*α*, IL-1*β*, and IL-6 were measured by qRT-PCR analysis (d). SE-EA treatments suppressed the expression of inflammatory genes. Values are mean ± standard deviation. # marks vs. the control group, ∗ marks vs. the LPS-stimulated group. ^∗^ *p* < 0.01, ^∗∗^ *p* < 0.05, and ^∗∗∗^ *p* < 0.001. ns: statistically not significant. *p* values were achieved using a one-way ANOVA analysis (Tukey method).

To determine the cytotoxic traits of SE-EA in the BV-2 microglial cells, the cells were inflicted with LPS (200 ng/mL) in the presence or absence of SE-EA at various concentrations (20, 100, and 200 *μ*g/mL), and an MTT assay was performed. The results indicate that cell viability was not affected, either at the evaluated concentrations of LPS alone or with SE-EA fractions for 20 h, nor was it found to be more than 80% (Figure 3(b)). Subsequently, to determine the intracellular ROS inhibitory effects of SE-EA, the intracellular NO released in BV-2 cells inflicted with LPS (200 ng/mL) in the presence or absence of each concentration of SE-EA was evaluated by a NO Griess reagent assay. As anticipated, LPS infliction significantly elevated the intracellular NO levels eight times higher than the untreated control cells. The SE-EA fractions exhibited a dose-dependent inhibition of NO activity by reducing the intracellular NO levels in LPS-inflicted BV-2 cells. More precisely, at the evaluated higher concentration of 200 *μ*g/mL, SE-EA markedly reduced the NO level nearly threefold more than the only LPS-treated cells.

#### 3.2.2. SE-EA Suppressed ROS Generation and Inflammatory Protein/mRNA Markers in LPS-Stimulated BV-2 Microglia Cells

Furthermore, to investigate the anti-ROS effects of SE-EA in BV-2 cells, the intracellular ROS generated during the physiological cellular process was detected using DCFDA dye and is quantified by measuring its fluorescence intensity to depict the ROS generation. The results of our study indicate that the SE-EA dose dependently suppresses DCF fluorescence intensity, wherein at the higher concentration of 200 *μ*g/mL, SE-EA reduces fluorescence intensity by 52.5 ± 7.2% compared to the control group (Spl.fig.  1A). Subsequently, the effects of SE-EA on mRNA and the protein expressions of heme oxygenase-1 (HO-1), a renowned ROS-defensive enzyme, whose downstream mediators were reported to promote the curbing of ROS generation, were investigated in LPS-inflicted BV-2 cells. The results of our study indicate that LPS-inflicted BV-2 microglial cells exhibit a mild rise in the levels of mRNA and protein expressions of HO-1, which is significantly elevated by SE-EA-treated cells in a dose-dependent manner. At the evaluated higher concentration of 200 *μ*g/mL, SE-EA exhibits threefold higher levels of mRNA and protein expressions of HO-1, compared to the LPS-treated group. Interestingly, the SE-EA- (200 *μ*g/mL) alone-treated cells express twofold higher levels of HO-1 expression than the LPS-treated group (Figure 3(a)).

To examine the anti-inflammatory potential of SE-EA, its modulatory effects on the proinflammatory mediators and cytokine level in LPS-stimulated BV-2 microglial cells were investigated (Figure 3(d)). The variations in the mRNA levels of proinflammatory cytokines (TNF-*α*, IL-1*β*, and IL-6) and proinflammatory mediators (iNOS and COX-2) were observed after 6 h, followed by LPS induction. The densitometric study of the bands reveals that the LPS-inflicted BV-2 cells significantly elevate the level of TNF-*α*, IL-1*β*, and IL-6 mRNA expressions sixfold, twofold, and twentyfold higher, respectively, than the control groups. Consequently, the iNOS and COX-2 mRNA expressions of LPS-inflicted cells were also found to increase by twofold and onefold, respectively, compared to those of the control group. Alternatively, SE-EA treatment dose-dependently suppressed the elevation of mRNA proinflammatory markers' expression. Precisely at the concentration of 200 *μ*g/mL, SE-EA suppressed the mRNA expressions of TNF-*α*, IL-1*β*, IL-6, iNOS, and COX-2 and nearly restored their levels proximal to the untreated control cells. This was further confirmed by the western analysis of the protein expression levels of iNOS and COX-2 (Figure 3(c)). As anticipated, the LPS-stimulated BV-2 cells exhibited 2.5 ± 0.3‐ and 7.5 ± 3.3‐fold increase compared to the control cells, which was effectively retaliated by SE-EA in a dose-dependent manner. At concentrations of 200 *μ*g/mL, SE-EA suppressed the protein expressions of iNOS and COX-2 with a fold change of 7.5 ± 3.3% and 2.5 ± 0.9%, respectively.

#### 3.2.3. SE-EA Attenuated the Functional Motor Deficits and Tyrosine Hydroxylase (TH) Depletion in a MPTP-Intoxicated PD-Like Mouse Model

**Figure 4:**
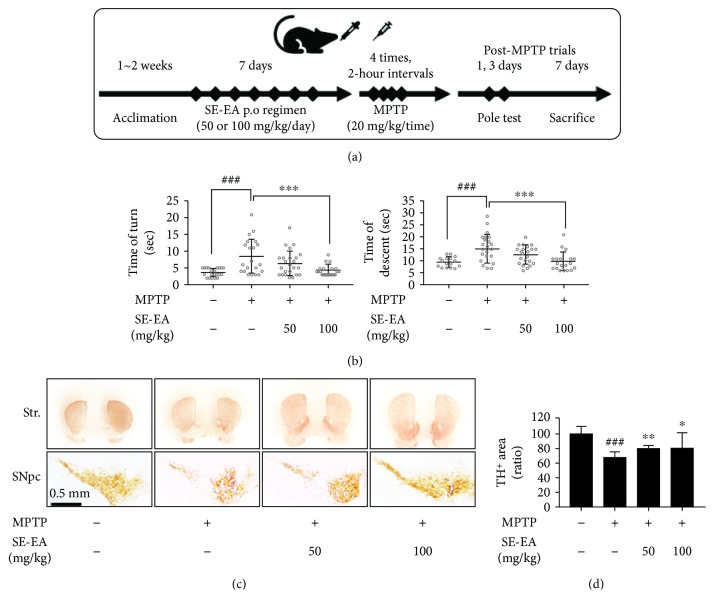
SE-EA-attenuated motor deficits and tyrosine hydroxylase depletion in MPTP-intoxicated PD-like mouse model. Animal experiment schedule is presented (a). Mouse coordination function was measured using a pole test. Each result is marked by dots and a thick bar is mean ± standard deviation (b). In IHC/DAB, the staining results indicate TH-positive neurons (substantia nigra pars compacta or SNpc) and its axon terminals (striatum or Str.). In the SNpc region, dopaminergic neurons stained by the IHC/DAB staining method and the TH-positive area were measured using ImageJ (c). In the graph, the relative TH-positive area's mean ± standard deviation is presented (d). ^###^ *p* < 0.001 vs. the control group and ^∗^ *p* < 0.01, ^∗∗^ *p* < 0.05, and ^∗∗∗^ *p* < 0.001 vs. LPS-stimulated group. *p* values were achieved using a one-way ANOVA analysis (Tukey method).

To evaluate the anti-PD-like protective effects of SE-EA, we employed an MPTP-induced PD-like mouse model. The mice were intoxicated using a four-time intraperitoneal administration of MPTP (20 mg/kg of bw) for a single day, with or without an SE-EA regimen (50 and 100 mg/kg/day) for a week (Figure 4(a)). The untreated (control) groups were evaluated regarding their functional motor deficits using a pole test. The results of our study indicate that the MPTP-induced mice required more time for the turn and descent test compared to the control group. This deleterious motor deficit was effectually ameliorated by SE-EA in a dose-dependent manner (Figure 4(b)). Furthermore, to evaluate the neuroprotective effects of SE-EA, the immunoreactivity of the TH-positive neurons in SNpc and STR was obtained through immunohistochemistry (IHC) (Figure 4(c)). The positive-stained TH neurons at SNpc were quantified, and their relative expressions are depicted in a representative graph (Figure 4(d)). According to SNpc and STR images of the brain section, MPTP-administered mice exhibit less TH-positive staining compared to the control groups, with a 40% reduction in the relative TH^+^ ratio at SNpc. The expression of the relative TH^+^ ratio was substantially elevated in SE-EA-treated mice, with a value of 20% more than the MPTP-inflicted mice, thereby exhibiting substantial protective effects of SE-EA on TH-immunopositive fibres in the striatum and the SNpc regions of mouse brains, proximal to the mice of the control group.

Overall, SE-EA substantially curbed by the neuroinflammatory cascades in microglial cells and thereby effectively attenuated the behavioural deficits in MPTP-induced PD-like animal models. Precisely as anticipated, the LPS infliction in the BV-2 microglial cells exhibited substantial elevation in the levels of NO production, proinflammatory cytokine (TNF-*α*, IL-1*β*, and IL-6), and mediator (iNOS and COX-2) expressions, which has been markedly reduced by SE-EA and thereby exhibits significant antineuroinflammatory potential. This is in accordance with the results of previous studies, in which the antineuroinflammatory potential of the evaluated extracts/candidates was validated by effectual suppression of the NO and proinflammatory mediator/cytokine levels in stimulated glial cells [45, 49, 50]. Subsequently, SE-EA was also found to elevate the HO-1 expressions in the stimulated glial cells, which may potentially contribute to its antineuroinflammatory and antioxidant potential. A recent study discussed the potential of HO-1 expressions as modulatory targets of neuroinflammation in neurodegenerative diseases [51]. Subsequently, several dietary antioxidants were also reported to upregulate HO-1 expression and exert substantial neuroprotective effects [52, 53]. In addition, it is also evident that the downstream effects by products of HO-1 were reported to exhibit antioxidant responses against ROS conditions [52, 54]. In accordance, the DCF-DA results of our study indicate that the physiological ROS generated in the glial cells are substantially reduced by the SE-EA. By confirming the antineuroinflammatory and anti-ROS potential of SE-EA, we further extended our study to investigate the anti-PD-like potential of SE-EA in the MPTP-intoxicated mouse model. Previous studies strongly suggest that MPTP intoxication significantly inflicts bradykinesia-like motor deficits in animals, which are determined by behavioural assays, such as the pole test [50, 55, 56]. Zhang et al. have reported that salidroside, a natural glucoside from *Rhodiola rosea* L., effectively improved the behavioural deficits in MPTP-induced PD mice, as observed by time of turn and time to reach floor in pole tests [57]. In accordance, the results of our study reveal severe behavioural deficits in MPTP-intoxicated mice with prolonged time latencies for the animals to turn and to reach the base. This was substantially attenuated by the SE-EA pretreatment and evidently improved the behavioural motor impediments. Subsequently, at the molecular level, the reduction of dopamine levels was substantially determined through the depletion of tyrosine hydroxylase- (TH-) positive fibres in the striatal SNpc regions of PD brains [58–60]. Accordingly, in our study, the MPTP-intoxicated mice exhibited significant DA neuronal loss in the striatal and SNpc regions with decreased TH^+^-stained neurons. Alternatively, SE-EA pretreatment effectually restored the levels of TH^+^-stained neurons in the striatal and SNpc regions, thereby exhibiting substantial neuroprotective potential by preventing a loss of dopaminergic neurons in PD progression.

### 3.3. Isolation and Investigations of the In Vitro Antioxidative and Anti-Inflammatory Potential of Irilin B

#### 3.3.1. Isolation and Characterization of the Bioactive Candidate Irilin B from SE-EA Fraction

To identify the potential bioactive candidate responsible for the above described protective effects of SE-EA, we performed an antioxidant activity- (i.e., DPPH assay-) guided partitioning and subsequently narrowed down and isolated Irilin B (L13-1) as a potential candidate. The schematic flow of the bioactivity guide isolation of Irilin B is depicted in Figure 2(a). Characterization of the purified compound, L13-1 (see Figure 2(a)), a pale-yellow amorphous powder (8 mg), was conducted by ESI-MS, ^1^H-NMR, and ^13^C-NMR analysis. ESI-MS were obtained using an LC-ESI mass spectrometer (Agilent 1100, Agilent Technologies, USA) in both negative and positive modes. ^1^H-NMR and ^13^C-NMR spectra were measured on a Jeol instrument (^1^H-NMR at 600 MHz, ^13^C-NMR at 150 MHz; JNM-ECA 600, Jeol, Japan) in DMSO-*d*6. The UV spectrum of the purified compound, L13-1 (Agilent, 1200 DAD, 190-400 nm, 20 nm step), displayed *λ*-maxima at 214 nm, 263 sh, 289 sh, and 338 sh (MeOH); 271 nm and 340 nm (+NaOAc); and 215 nm, 271 nm, 317 nm, and 368 nm (+AlCl_3_), which are characteristics of flavonoid-isoflavones. To identify the compound L13-1, further analyses were performed using an electrospray ionization- (ESI-) mass spectrometry (MS) and a NMR spectroscopy. The ESI-MS results, m/z 301.1 [M+H]^+^ and m/z 299.1 [M-H]^+^, indicate that the molecular weight of compound L13-1 is 300 Da (C_16_H_12_O_6_) (Figure 2(c)). Compound L13-1 was finally identified as an isoflavone, Irilin B (5,7,2-trihydroxy-6-methoxy-isoflavone), based on the assignment of the proton and carbonyl resonances observed in the spectra of ^1^H-NMR, ^13^C-NMR (Figure 2(c)), HMBC, and ^1^H-^1^H COSY (Figure 2(c)). ^1^H-NMR (600 MHz, DMSO-*d*6) *δ* ppm: 3.87 (1H, d, 3-H), 6.47 (1H, d, 8-H), 8.07 (1H, d, 2-H), 6.90 (1H, d, 3′-H), 7.23 (1H, m, 4′-H), 6.89 (1H, d, 5′-H), and 7.23 (1H, s, 4′-H) (Figure 2(c)); ^13^C-NMR (150 MHz, DMSO-*d*6) *δ* ppm: 157.0 (2-C), 122.0 (3-C), 182.6 (4-C), 156.8 (5-C), 133.0 (6-C), 159.0 (7-C), 95.2 (8-C), 155.0 (9-C), 106.4 (10-C), 119.3 (1′-C), 155.9 (2′-C), 117.1 (3′-C), 130.9 (4′-C), 120.7 (5′-C), 132.7 (6′-C), and 60.7 (OCH_3_) (Figure 2(c)).

#### 3.3.2. In Vitro Antioxidative and Anti-Inflammatory Effects of Irilin B in LPS-Stimulated BV-2 Microglial Cells

**Figure 5:**
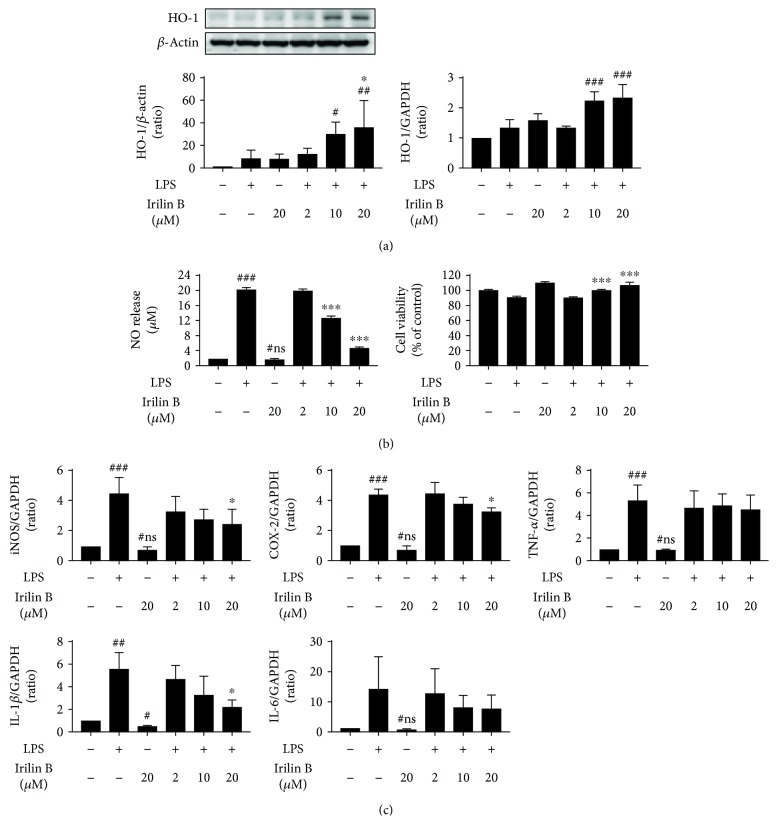
Effects of Irilin B in molecular level alterations of proinflammatory cytokines/mediators and antioxidant biomarker. A major flavonoid of SE-EA, Irilin B (20, 10, and 20 *μ*M) was treated on LPS-stimulated BV-2 microglial cells. Cells were incubated for 20 hours in a CO_2_-supplied incubator, and the HO-1 expression levels were measured using a western blot and qRT-PCR (a). Nitric oxide (NO) releases were measured by Griess reagent assay, and cell viabilities were assayed by using MTT reagent (b). Including iNOS and COX-2, proinflammatory cytokines, TNF-*α*, IL-1, and IL-6 expression levels were analysed using the qRT-PCR method (c). # marks vs. the control group, ∗ marks vs. the LPS-stimulated group. ^∗^ *p* < 0.01, ^∗∗^ *p* < 0.05, and ^∗∗∗^ *p* < 0.001. ns: statistically not significant. *p* values were achieved using a one-way ANOVA analysis (Tukey method).

To further confirm the bioactive potential of the isolated Irilin B, we evaluated its *in vitro* antioxidative and anti-inflammatory potential in LPS-stimulated BV-2 microglial cells, as previously described. Initially, the cell viability of the BV-2 cells was evaluated to confirm the potential toxicity of Irilin B, which may have possibly been incurred during the isolation process. At the evaluated concentrations (2, 10, and 20 *μ*M) of Irilin B and LPS (200 ng/mL), the stimulation did not exhibit substantial toxicity and indeed exhibited more than 80% of cell viability. However, LPS infliction for 20 h elevated the NO release in BV-2 microglial cells nearly five times more than the untreated control cells and is effectually curbed by the Irilin B in a dose-dependent fashion (Figure 5(b)). Indeed, at a concentration of 20 *μ*M, Irilin B exhibited nearly a fourfold decrease in NO levels compared to the LPS-stimulated cells. This was in accordance with the anti-inflammatory effects of Irilin B, whereby the elevated expressions in the levels of proinflammatory mRNA markers (i.e., TNF-*α*, IL-1*β*, IL-6, iNOS, and COX-2) in LPS-stimulated cells were substantially suppressed by Irilin B (Figure 5(c)). More precisely, at the evaluated higher concentrations of 20 *μ*M, Irilin B suppressed the levels of proinflammatory mRNA expressions twice as much as the LPS-treated group. On the other hand, Irilin B was also found to exhibit potential antioxidative potential, similar to SE-EA. The elevated ROS levels relatively quantified by an increase in DCF fluorescence intensity in the physiological condition of BV-2 cells were substantially curbed by the Irilin B dose, and a twentyfold decrease in DCF intensity was observed for the 20 *μ*M concentration, compared to the LPS-inflicted cells (supplementary file-2). This was subsequently confirmed by a dose-dependent elevation of the mRNA and protein expressions of heme oxygenase-1 (HO-1), the ROS-defensive enzyme, thereby potentially retaliating against the ROS generated in the LPS-stimulated BV-2 cells (Figure 5(a)).

Thus, based on the in vitro antioxidant activity-guided isolation of the bioactive candidate, among various candidates/fractions of SE-EA, the compound L13-1, Irilin B, exhibited enhanced antioxidative activity and is thereby identified as a potential bioactive candidate of SE-EA. This is in accordance with the results of previous studies, in which the Irilin B, isolated from Iris songarica Schrenk, exhibited substantial antioxidant activity by inhibiting ROS generation in phorbol myristate acetate- (PMA-) induced HL-60 leukemia cell lines. Subsequently, the purity and characterization of the isolated Irilin B were further confirmed using chromatographic and NMR techniques. Irilin B, isolated as a pale-yellow amorphous powder, exhibited the traits of flavonoid-isoflavones and is confirmed as the 5,7,2-trihydroxy-6-methoxy-isoflavone, with a molecular weight of 300 Da (C_16_H_12_O_6_). Nevertheless, we have identified and reported the existence of Irilin B in SE-EA, which could potentially contribute to the antioxidant and anti-inflammatory potential of SE-EA in scopolamine-induced amnesic mice [29]. In accordance, in this study, the antineuroinflammatory and antioxidant potential of the isolated Irilin B was demonstrated by anti-ROS and anti-inflammatory activities in LPS-stimulated BV-2 cells. This indicates that Irilin B can potentially contribute to the anti-PD-like activity of SE-EA in MPTP-intoxicated mice.

## 4. Conclusions

This study demonstrates that *Salicornia europaea* L. extract and its bioactive compound, Irilin B, exert antineuroinflammatory and anti-ROS effects through the inhibition of several proinflammatory mediators and oxidative stress markers in the stimulated microglial cells. Moreover, the anti-PD-like potential of SE-EA was also evidently demonstrated in the MPTP-intoxicated mouse model. This study, for the first time, demonstrated the antineuroinflammatory potential of Irilin B. However, further mechanistic studies are required to develop SE-EA and Irilin B as effectual therapeutics to target inflammation and oxidative stress-mediated neurodegenerative complications.

## Figures and Tables

**Table 1:** The mRNA primers used are the following. Accession numbers of the gene bank are specified, and expected product sizes are listed.

Target gene		Sequence (5′->3′)	Accession no.	Product size (bp)
iNOS	Forward	TGAAGAAAACCCCTTGTGCT	NM_010927	100
	Reverse	TTCTGTGCTGTCCCAGTGAG		

COX2	Forward	CAAGACAGATCATAAGCGAGGA	NM_011198	107
	Reverse	GGCGCAGTTTATGTTGTCTGT		

TNF-*α*	Forward	CCACCACGCTCTTCTGTCTAC	NM_013693	103
	Reverse	AGGGTCTGGGCCATAGAACT		

IL-1*β*	Forward	TGTGAAATGCCACCTTTTGA	NM_008361	94
	Reverse	GGTCAAAGGTTTGGAAGCAG		

IL-6	Forward	TGATGCACTTGCAGAAAACA	NM_031168	109
	Reverse	ACCAGAGGAAATTTTCAATAGGC		

GAPDH	Forward	AAGGGCTCATGACCACAGTC	NM_001289726	160
	Reverse	TTCAGCTCTGGGATGACCTT		

## Data Availability

The data used to support the findings of this study are included within the article.

## Supplementary Materials

Supplementary Materials Supplementary Figure 1: SE-EA (20,100, and 200 *μ*g/mL) and Irilin B (2, 10, and 20 *μ*M) were treated onto BV-2 microglial cells, and the cells were incubated 20 hours. Later, cellular reactive oxygen species (or ROS) productions were measured by DCF-DA assay (A). Cells were incubated 20 hours post-Irilin B/LPS cotreatment in a CO_2_-supplied incubator. In western blot, iNOS and COX-2 protein expression levels were shown. Each of the bands was analysed by ImageJ, and the relative intensity of the bands was presented in graphs (B). Values are mean ± standard deviation. # marks vs. control group, ∗ marks vs. LPS-stimulated group. ^∗^ *p* < 0.01, ^∗∗^ *p* < 0.05, and ^∗∗∗^ *p* < 0.001. ns: statistically not significant. *p* values were achieved by using one-way ANOVA analysis (Tukey method). Supplementary Figure 2: SE-EA (20, 100, and 200 *μ*g/mL) and LPS (200 ng/mL) were treated onto BV-2 microglial cells, and the cells were incubated 30 minutes. Cells were lysed by RIPA buffer immediately. As presented in the western blot image, LPS treatment increased phosphorylation of MAPK signaling molecules (A). Supplementary Figure 3: SE-EA (20, 100, and 200 *μ*g/mL) and LPS (200 ng/mL) were treated onto BV-2 microglial cells, and the cells were incubated for 2 hours. Cytosolic and nucleic protein samples were collected; we confirmed Nrf2 protein translocation by western blot (A). Long-term exposures (20 hours) of SE-EA and LPS were observed too (B). LPS treatment was induced by phosphorylation of IRF3 proteins (C).
