# Supplementary material for: The Ameliorative Effects of the Ethyl Acetate Extract of Salicornia europaea L. and Its Bioactive Candidate, Irilin B, on LPS-Induced Microglial Inflammation and MPTP-Intoxicated PD-Like Mouse Model
Source: Oxid Med Cell Longev. 2019 Jul 9;2019:6764756. doi: 10.1155/2019/6764756 (PMC6652089; doi:10.1155/2019/6764756)
Supplement: Supplementary Materials — Supplementary Figure 1: SE-EA (20,100, and 200 μg/mL) and Irilin B (2, 10, and 20 μM) were treated onto BV-2 microglial cells, and the cells were incubated 20 hours. Later, cellular reactive oxygen species (or ROS) productions were measured by DCF-DA assay (A). Cells were incubated 20 hours post-Irilin B/LPS cotreatment in a CO2-supplied incubator. In western blot, iNOS and COX-2 protein expression levels were shown. Each of the bands was analysed by ImageJ, and the relative intensity of the bands was presented in graphs (B). Values are mean ± standard deviation. # marks vs. control group, ∗ marks vs. LPS-stimulated group. ∗ p < 0.01, ∗∗ p < 0.05, and ∗∗∗ p < 0.001. ns: statistically not significant. p values were achieved by using one-way ANOVA analysis (Tukey method). Supplementary Figure 2: SE-EA (20, 100, and 200 μg/mL) and LPS (200 ng/mL) were treated onto BV-2 microglial cells, and the cells were incubated 30 minutes. Cells were lysed by RIPA buffer immediately. As presented in the western blot image, LPS treatment increased phosphorylation of MAPK signaling molecules (A). Supplementary Figure 3: SE-EA (20, 100, and 200 μg/mL) and LPS (200 ng/mL) were treated onto BV-2 microglial cells, and the cells were incubated for 2 hours. Cytosolic and nucleic protein samples were collected; we confirmed Nrf2 protein translocation by western blot (A). Long-term exposures (20 hours) of SE-EA and LPS were observed too (B). LPS treatment was induced by phosphorylation of IRF3 proteins (C). [file 6764756.f1.docx]

**Supplementary figure 1.
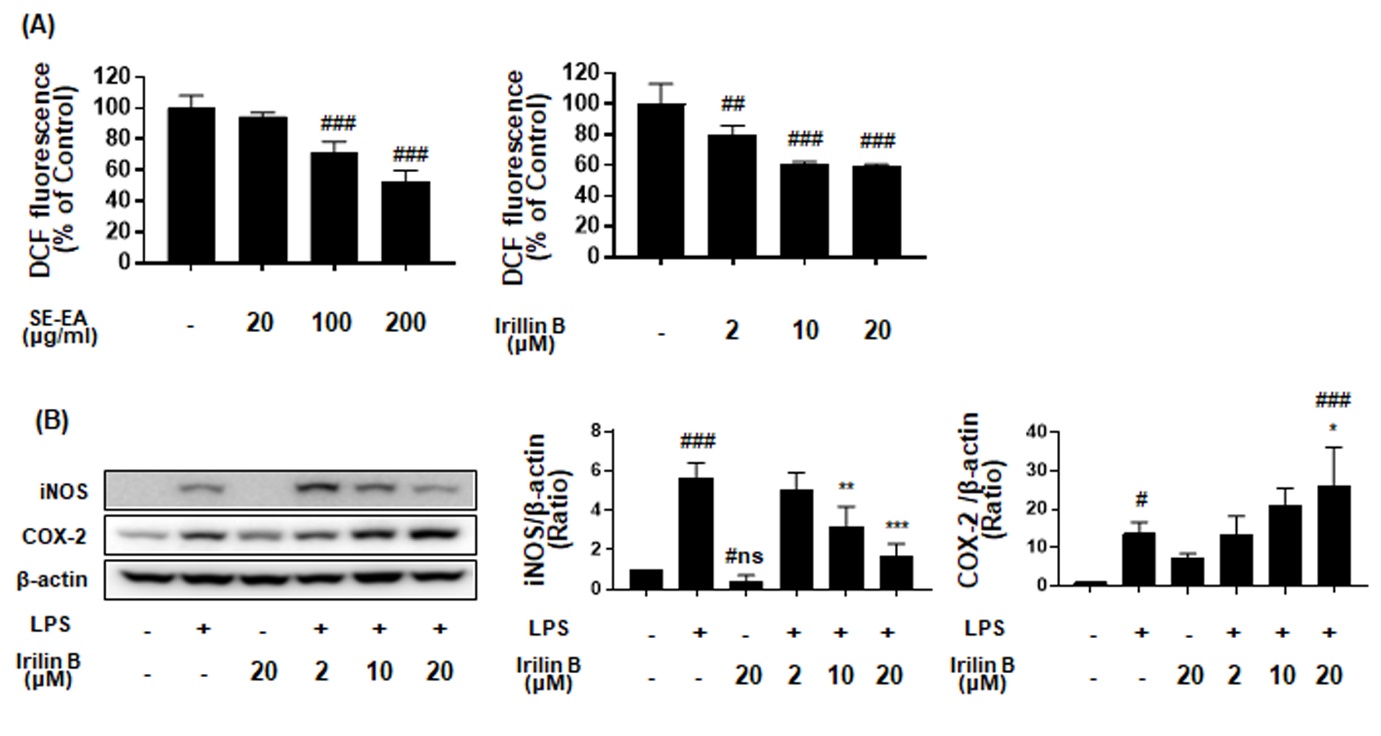
**

**Supplementary figure 2.**


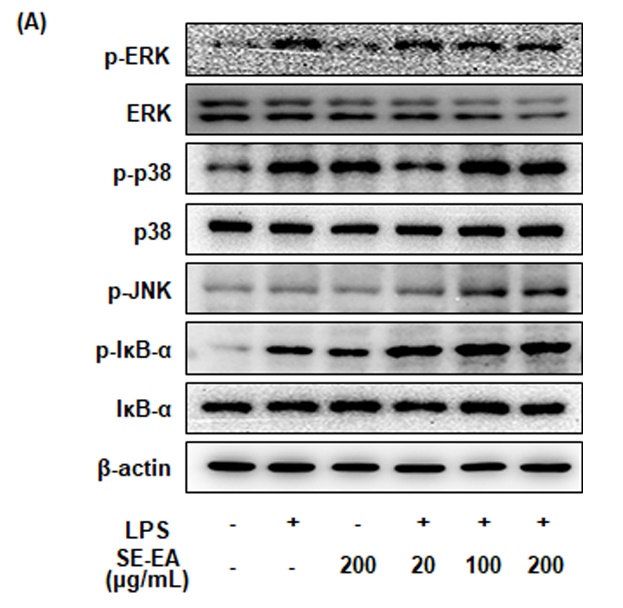


**Supplementary figure 3.
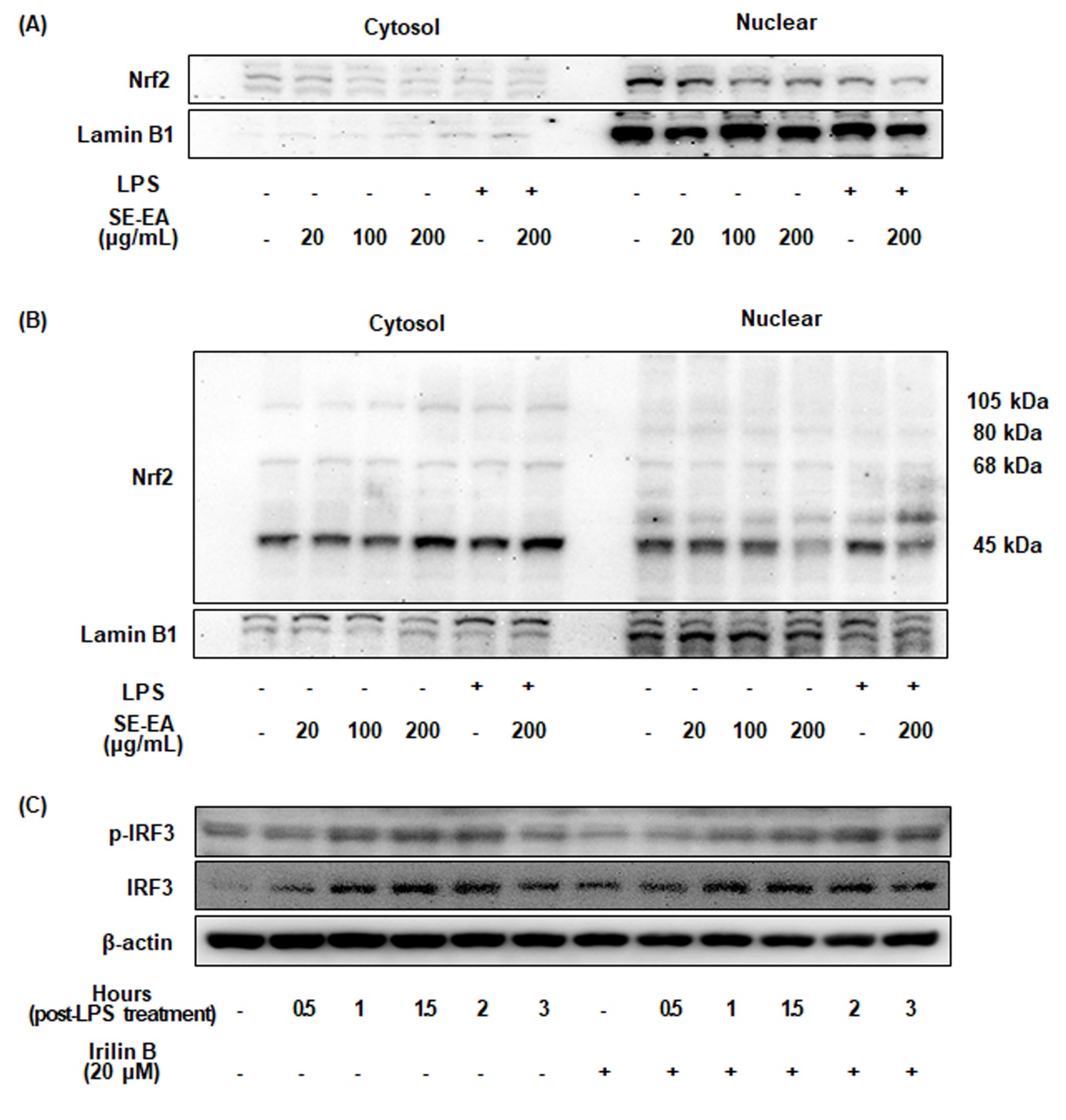
**

**Supplementary figure 1**.

SE-EA (20,100 and 200 μg/mL) and irilin B (2,10 and 20 μM) was treated onto BV-2 microglial cells and cells were incubated 20 hours. Later, cellular reactive ozygen species (or ROS) productions were measured by DCF-DA assay (A). Cells were incubated 20 hours post irillin B/LPS co-treatment in CO2 supplied incubator. In western blot, iNOS and COX-2 protein expression levels were shown. Each of bands were analyzed by imageJ and relative intensity of bands were presented in graphs (B). Values are mean ± standard deviation. ‘#’ marks vs. control group, ‘*’ marks vs. LPS-stimulated group. *p < 0.01, **p < 0.05, ***p < 0.001. ‘ns’ is abbreviation of statistically no significant. P values were achieved by using one-way ANOVA analysis (Tukey method).

**Supplementary figure 2**.

SE-EA (20,100 and 200 μg/mL) and LPS (200 ng/mL) was treated to BV-2 microglial cells and cells were incubated 30 minutes. Cells were lysed by RIPA buffer immediately. As presented in western blot image, LPS treatment increased phosphorylation of MAPKs signaling molecules (A).

**Supplementary figure 3**.

SE-EA (20,100 and 200 μg/mL) and LPS (200 ng/mL) was treated to BV-2 microglial cells and cells were incubated for 2 hours. Cytosolic and nucleic protein samples were collected, we confirmed Nrf2 protein translocation by western blot (A). Long-term exposures (20 hours) of SE-EA and LPS were observed too (B). LPS treatment was induced phosphorylation of IRF3 proteins (C).
